# Lameness prevalence and management practices on Irish pasture-based dairy farms

**DOI:** 10.1186/s13620-022-00221-w

**Published:** 2022-06-08

**Authors:** N. Browne, C. D. Hudson, R. E. Crossley, K. Sugrue, E. Kennedy, J. N. Huxley, M. Conneely

**Affiliations:** 1 Teagasc, Animal & Grassland Research and Innovation Centre, Moorepark, Fermoy, Co. Cork, Ireland; 2 School of Veterinary Medicine and Science, University of Nottingham, Loughborough, UK; 3 Animal Production Systems Group, Department of Animal Sciences, Wageningen University & Research, Wageningen, the Netherlands; 4 Massey University, School of Veterinary Science, Palmerston North, New Zealand

**Keywords:** Lameness, Dairy cow, Infrastructure, Management, Welfare

## Abstract

**Background:**

Lameness is a painful disease, which negatively impacts dairy cow production and welfare. The aim of this observational study was to determine herd lameness prevalence, describe current lameness management practices and identify the presence of established risk factors for lameness on Irish pasture-based dairy farms. Farms were visited once during grazing (99 farms) and again during housing (85 farms). Lameness scoring was carried out at each visit (AHDB 0–3 scale); cows were classified as lame if they scored two or three. Farm management practices and infrastructure characteristics were evaluated via farmer questionnaires and direct measurements of farm infrastructure.

**Results:**

Median herd-level lameness prevalence was 7.9% (interquartile range = 5.6 – 13.0) during grazing and 9.1% (interquartile range = 4.9 – 12.0) during housing; 10.9% of cows were lame at a single visit and 3.5% were lame at both visits (chronically lame or had a repeat episode of lameness). Fifty-seven percent of farmers were not familiar with lameness scoring and only one farm carried out lameness scoring. Only 22% of farmers kept records of lame cows detected, and 15% had a lameness herd health plan. Twenty-eight percent of farmers waited more than 48 h to treat a lame cow, and 21% waited for more than one cow to be identified as lame before treating. Six percent of farmers carried out routine trimming and 31% regularly footbathed (> 12 times per year). Twelve percent put severely lame cows in a closer paddock and 8% stated that they used pain relief to treat severely lame cows. Over 50% of farms had at least one cow track measurement that was classified as rough or very rough, and cow tracks were commonly narrow for the herd size. On 6% of farms, all cubicle beds were bare concrete (no matting or bedding) and on a further 6% of farms, there was a combination of cubicles with and without matting or bedding. On 56% of farms, all pens contained less than 1.1 cubicles per cow and on 28% of farms, a proportion of pens contained less than 1.1 cubicles per cow.

**Conclusions:**

Overall, this study identified infrastructure and management practices which could be improved upon. The comparatively low lameness prevalence demonstrated, compared to fully housed systems, also highlights the benefits of a pasture-based system for animal welfare; however, there remains scope for improvement.

## Background

Lameness is a result of pain [1, 2] and is, therefore, a major animal welfare issue and an on-going concern within the dairy industry. Lameness has a negative economic impact due to reduced milk yields [3, 4] and reproductive ability [5–7], increased culling rates and replacement costs [8–10], and increased treatment [11] and labour costs [12]. Economic costs also result from discarding milk due to antibiotic use [9, 10], reocurring lameness cases [9] and implementing lameness prevention methods [13]. Lameness also has a negative environmental impact due to increased greenhouse gas emissions [14, 15].

Reported lameness prevalence has generally been higher in housed systems and lower in pasture-based systems [16]. Average herd-level prevalence in pasture-based systems has been reported between 3.7% in Swedish dairy farms [17] and 35% in small-scale Brazilian dairy farms [18]. Whereas in housed systems, average herd-level prevalence has been reported between 9.6% and 55% [19, 20]. Access to pasture is thought to be beneficial to animal health and wellbeing, allowing cows more opportunity to exhibit normal behaviours [21]. Depending on conditions, pasture provides an optimal walking surface for improved mobility [22] and a soft surface and space for cows to transition between bouts of standing and lying [23].

In Irish pasture-based herds, where cows are generally out to pasture for the majority of the year and housed for approximately 4.5 months during the winter period [24], average herd-level lameness prevalence has ranged from 5.9% towards the end of the grazing period [25] to 14.6% during the breeding season [26]. Although lameness prevalence during the grazing period has previously been reported on Irish dairy farms, limited studies have examined the prevalence of lameness during both the grazing and housing periods, and the transition between the two. Lameness prevalence in Ireland has also only been reported prior to quota removal; therefore, prevalence may have altered since farmers have had the opportunity for farm expansion. Furthermore, no studies to date have reported how lameness status at cow-level changes between the housing and grazing periods in Irish systems. Determining if the same cows remain lame or are recurrently lame during both periods will help with understanding the dynamics of lameness in part-housed, part-grazed dairy cows.

Lameness prevention methods, as well as early detection and treatment, are fundamental to effective lameness control programs [27–30]. However, very limited information currently exists on current lameness control strategies in Ireland. O’Connor et al. [25] revealed that approximately half of farmers in Ireland footbathed at least once per year; however, no details were provided on the footbathing protocols used. Additionally, limited data exists regarding the use of routine hoof trimming to prevent lameness and the use of lameness scoring to detect lame cows. Identifying the strategies Irish dairy farmers use to control lameness will help pinpoint areas for improvement, and deliver a focus to farmers, advisors and veterinarians regarding the best strategies to reduce lameness prevalence in Ireland.

It is also essential to determine the current general management practices and infrastructure characteristics on Irish dairy farms. This information will provide details on where improvements are needed, and help to identify which areas may pose a risk of lameness. As part of a survey-based study, Boyle et al. [31] reported that there was a lack of investment in cow tracks, handling facilities and housing in Irish pasture-based dairy herds as farms expanded, with more investment directed towards milking facilities. Although a small amount of information is available on current farm infrastructure in Ireland [31], this information was based on farmer surveys, as opposed to direct measurements on farm by external observers.

The aims of this study were to determine the herd-level lameness prevalence during both grazing and housing periods on Irish pasture-based dairy farms, and evaluate cow-level changes in lameness status and lameness scores across visits. A further aim was to identify current management practices and infrastructure in place on Irish dairy farms. This study ultimately aims to deliver useful knowledge to the dairy industry regarding aspects of lameness management where improvement is needed, and to provide direction for future research.

## Methods

This study was part of a larger project investigating welfare in pasture-based dairy herds [32, 33]. For full details of the methods used in this study, see Browne et al. [33].

In brief, herds were randomly selected from a list of dairy farms provided by the Irish Cattle Breeding Federation (ICBF; Bandon, Co. Cork, Ireland), who allowed Teagasc access to their data. Selection criteria included: herd size between 30 and 250 cows, located in the seven counties with the highest number of dairy cows, no further than two hours from Teagasc Moorepark and willingness to participate in the study. Based on a simulation-based power calculation, 100 farms was the target sample size.

One hundred and two (99 included in the analyses) Irish spring-calving, pasture-based dairy farms were visited between April and September 2019 during the grazing period, and 87 (85 included in the analyses) of these farms were revisited between October 2019 and February 2020 during the housing period. The main reason for the withdrawals at the housing visit was cows being close to calving. At each visit the entire milking herd was lameness scored using a four-point scale ranging from zero to three [34] and a proportion of each herd was body condition scored [35]. All scorers undertook training in lameness scoring and body condition scoring. Interobserver reliability, using weighted kappa coefficients, was carried out for lameness scoring and body condition scoring at the beginning of both the grazing visits and housing visits; all interobserver agreement were greater than 0.7. Hoof lesions were recorded for up to 20 cows identified as lame (lameness score [LS] 2 and LS3). This data is the subject of a separate publication (Browne et al., unpublished). Additional cow-level information (production data, calving data, breed and genetic profile) was also provided by the Irish Cattle Breeding Federation for each herd enrolled in the study.

A structured questionnaire was undertaken with the farmer at both the grazing visit and housing visit to identify farm characteristics and management practices, including methods for controlling lameness. Direct infrastructure measurements were also recorded on each farm in the milking parlour and collecting yard, in all pens used by dairy cows and on cow tracks. Cow track measurements were taken on the track in use on the day of the grazing visit; at the estimated half-way point between the collecting yard and paddock, the end-point of this track and the paddock gateway. Cow track measurements were also taken in the segment between the collecting yard entrance and fifty-metres along all tracks used by cows. The questionnaires, categorical scales used as part of the infrastructure measurements and further details on measurements taken are available to view as supplementary material [36].

### Statistical analysis

All data were analysed using R software version 3.3.1 (R Core Team, Vienna, Austria). Three farms from the grazing period and two farms from the housing period were not included in the analyses due to operating an automatic milking system or milking once per day. These farms were excluded as they were not considered to be representative of typical Irish dairy farms. These farms were also managed differently, so some measurements would not have been possible (e.g. parlour and collecting yard measurements). The final dataset consisted of 11,213 lameness scores (LS) from 99 farms (grazing period) and 8,995 LS from 85 farms (housing period).

Cows were categorised into lame (LS2 and LS3) and non-lame (LS0 and LS1) at each visit. Herd-level lameness prevalence was calculated for both the grazing and housing periods, defined as the number of lame cows divided by the total number of cows scored in the herd. Similarly, herd-level prevalence of severely lame cows (LS3 only) was calculated. For farms visited during both periods, lameness prevalence between the grazing and housing periods was compared using a t-test (normally distributed data) or the Wilcoxon test (non-normally distributed data). The difference in the proportion of each LS between periods was also compared using this method.

Cows that were lameness scored during both the grazing and housing periods were classified into four categories; no lameness (not lame at grazing or housing), became lame (not lame at grazing but lame at housing), recovered (lame at grazing but not housing) and remained lame (lame at both grazing and housing). The unit change in LS between the grazing and housing periods was also calculated. Descriptive statistics were calculated to summarize herd-level data gathered from the farmer questionnaires (milking practices and lameness detection, prevention and treatment methods) and infrastructure measurements (winter housing, cow tracks and milking facilities).

## Results

### Farm and cow characteristics

The median farmer-reported herd size across the 99 farms was 116 cows (interquartile range [IQR] = 81 – 156), with a median increase in herd size of 21% (IQR = 0 – 35) in the last five years. The median grazing platform size was 40 hectares (IQR = 29 – 52), with a median stocking rate of 2.9 cows per hectare (IQR = 2.3 – 3.5) and a median grazing season length of 252 days (IQR = 238 – 274). The median parity of cows was 3 (IQR = 2 – 5), calving interval was 369 days (IQR = 354 – 388) and 305-day yield was 6638 kg per cow (IQR 5750 – 7597). Seventy-two percent of cows were purebreds (51% Holstein–Friesian) and 28% were crossbreeds. The median body condition score during the grazing and housing period was 3 (IQR 3 – 3.25) and 3.25 (IQR = 3 – 3.5), respectively.

### Herd-level lameness prevalence

**Fig. 1:**
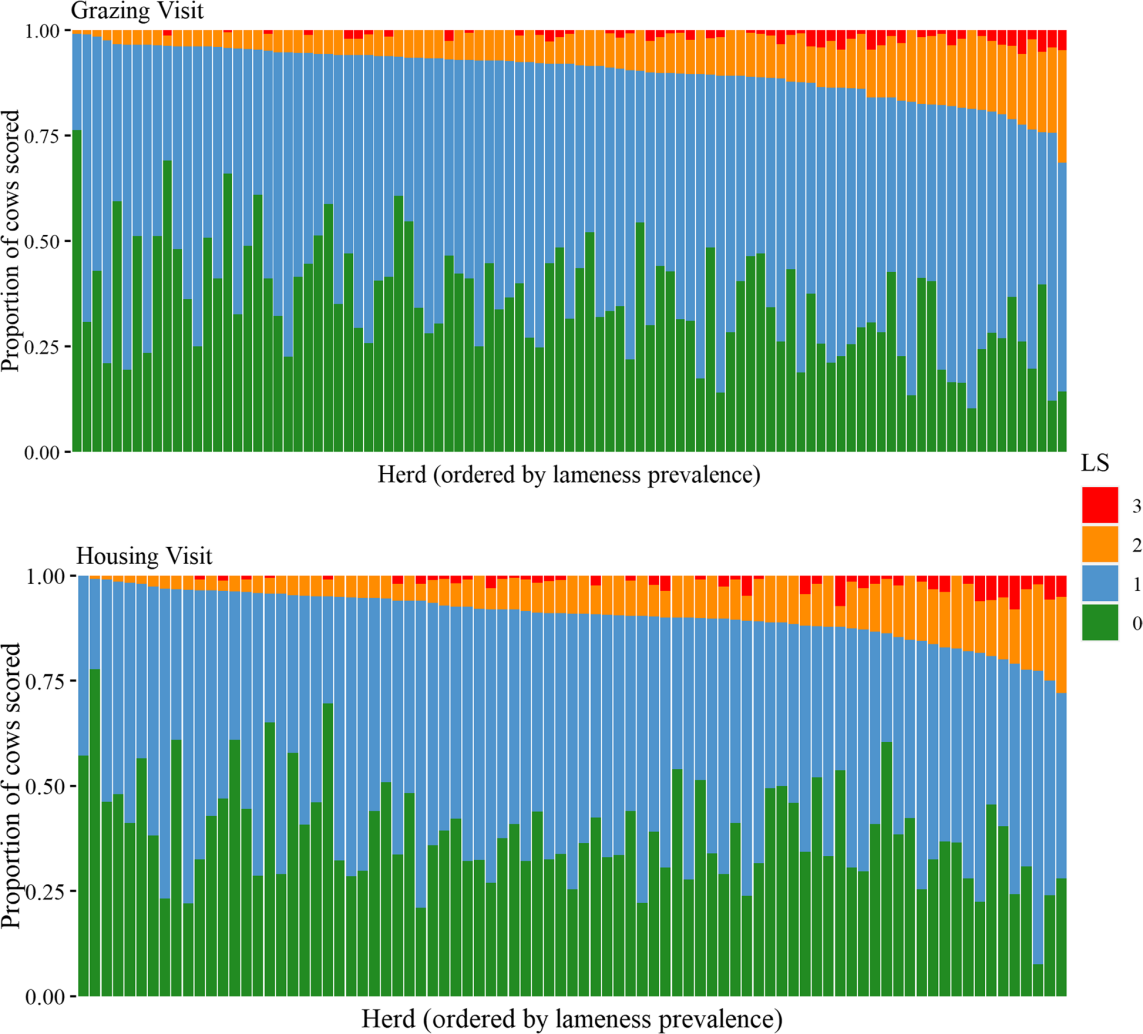
Proportion of each lameness score, ordered by lameness prevalence (LS2 and LS3), across 99 spring-calving, pasture-based herds during the grazing period (April 2019 – September 2019) and in 85 of these herds during the housing period (October 2019 – February 2020). Each bar represents one farm

The distribution of LS across each farm is shown in Fig. 1. The median herd-level lameness prevalence (LS2 and LS3) was 7.9% (IQR = 5.6—13.0) during the grazing period and 9.1% (IQR = 4.9 – 12.0) during the housing period. The median herd-level prevalence of severely lame cows (LS3) was 0.7% (IQR = 0.0—1.9) during the grazing period and 0.8% (IQR = 0.0—2.0) during the housing period.

There was no significant difference (*P* = 0.497) in lameness prevalence between visits for farms that were visited during both the housing and grazing periods (*n* = 85). There was, however, a small but statistically significant difference (*P* = 0.047) between the proportion of cows scored LS0 during grazing (35.5%) and housing (38.8%). There was no significant difference in proportions of cows scored LS1 (*P* = 0.085), LS2 (*P* = 0.179) or LS3 (*P* = 0.430) between the grazing and housing periods.

### Change in lameness status and lameness score

**Fig. 2:**
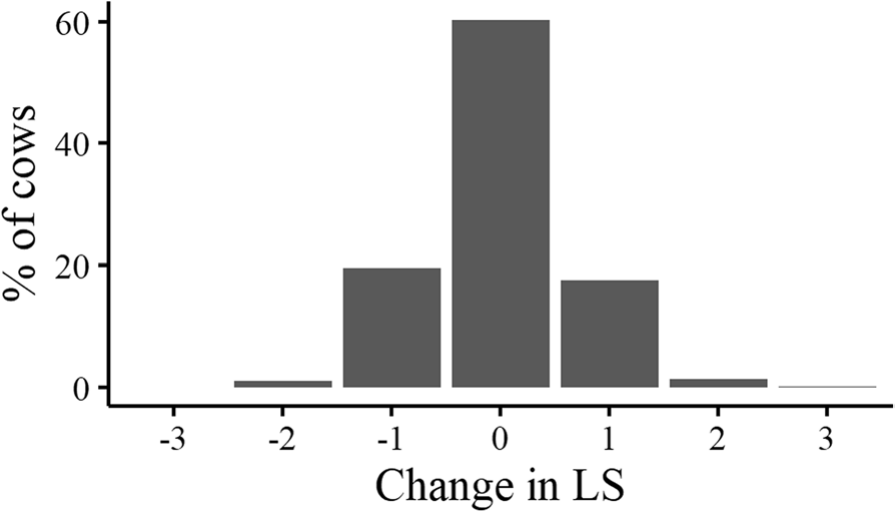
Percentage of cows for each unit change in lameness score between the grazing and housing periods for 8,676 cows from 85 spring-calving, pasture-based herds that were lameness scored during both the grazing (April 2019 – September 2019) and housing (October 2019 – February 2020) periods. Zero represents cows that had the same lameness score during both the grazing and housing periods, a negative value represents a decrease in lameness score and a positive value represents an increase in lameness score

A total of 8,676 cows were scored at both the grazing and housing visits; of these, 1,243 cows (14.4%) were lame at a minimum of one visit (Table 1). Of those cows that were lame during the grazing visit (778 cows), 305 (38.9%) remained lame at the housing visit and 473 (61.1%) recovered from lameness. Of those cows that were LS3 at grazing (81 cows), 50 (62.7%) remained lame at housing, whereas for cows that were LS2 at grazing (697 cows), 255 (36.6%) remained lame at housing. Of all cows scored (8676 cows), 1651 (19%) had an increase in LS, 1799 (21.7%) had a reduction and 5226 (60.2%) had the same LS during both the grazing and housing period (Fig. 2).

**Table 1:** Change in lameness status for 8,676 cows from 85 spring-calving, pasture-based herds that were lameness scored during both the grazing (April 2019 – September 2019) and housing (October 2019 – February 2020) periods. Lameness was defined as LS2 and LS3 on the Agricultural and Horticultural Development Board four-point scale

Description^a^	Lame at grazing	Lame at housing	Frequency	%
No lameness	No	No	7433	85.7
Became lame	No	Yes	465	5.4
Recovered	Yes	No	473	5.5
Remained lame	Yes	Yes	305	3.5

^a^No lameness = not lame at grazing or housing; Became lame = not lame at grazing but lame at housing; Recovered = lame at grazing but not housing; Remained lame = lame at both grazing and housing

### Lameness detection methods

Forty-three percent of farmers said they were familiar with lameness scoring; however, only one farm carried out lameness scoring. That farm lameness scored three times per year using a 0–3 scoring system. Only one farm used technology to detect lameness, using a neck-based accelerometer. Ninety-nine percent of farmers said they detected lameness through visual inspection (i.e. watching cows as they walk, not through formal lameness scoring), with one farmer saying they used no methods to detect lameness in their herd. Twenty-two percent of farmers kept records of lame cows they detected.

### Lameness prevention methods

Fifteen percent of farmers had a herd health plan that included lameness management protocols. Of these, 12% were created in conjunction with the farmer’s veterinarian and 3% were created by only the farmer. Six percent of farmers routinely trimmed the whole herd; of these, one farm routinely trimmed twice per year and five farms trimmed once per year. Of those that routinely trimmed, half trimmed both the front and back hooves and half trimmed the back hooves only. Eighty-three percent of routine trimming was carried out by a professional hoof trimmer and 17% by the farmer.

Thirty-one percent of farmers used preventative footbathing regularly (> 12 times per year), 20% irregularly (≤ 12 times per year), 5% used footbathing only if required and 43% never used preventative footbathing (percentages may not total 100% due to rounding). Of farms that carried out footbathing, 67% used a single product in their footbath and 33% used a combination of different products in their footbathing routine. The most common product used was copper sulphate (54% of farms that footbathed), followed by formalin (35%) and an organic acid and tea-tree solution (33%). The footbath product was changed after a median of 228 cows (IQR = 168—325) across farms. Of farms that carried out footbathing, 6% cleaned the cows’ hooves using a pre-wash footbath and 39% cleaned the cows’ hooves with a hose prior to footbathing. Eighty percent of footbaths used were less than three metres in length.

### Lameness treatment methods

According to the farmers, a median of 10% (IQR = 6—20) of each herd was treated for lameness in the last year. On 38% of farms, lameness treatment was completed by the farmer, 32% by a professional trimmer and 26% by a combination. Farmers would call a veterinary practitioner to treat a lame cow on 61% of farms; of these, 5% would request examination by a veterinary practitioner for all lame cows and 95% for severely lame cows, cows that do not recover or cows that could not be effectively treated by themselves or a trimmer.

Forty-nine percent of farmers aimed to treat cows within 24 h of detecting they were lame, 24% within 48 h, and 28% waited more than 48 h. Twenty-one percent of farmers waited for a number of cows to be lame before treating. On these farms, the median number of cows that needed to be lame before any were treated was 2.5 cows (IQR = 2.0 – 3.4). For a mildly lame cow, 4% of farmers said they would put the cow in a closer paddock and 1% would put the cow on once-a-day milking, whereas, for a severely lame cow, 12% of farmers would put the cow in a closer paddock and 4% would put the cow on once-a-day milking. Eleven percent provided antibiotics, 3% pain relief and 4% a form of unspecified medication to a mildly lame cow, whereas, for a severely lame cow, 23% provided antibiotics, 8% pain relief, and 8% a form of unspecified medication.

Nine percent of farmers used an antibiotic footbath as a treatment for digital dermatitis. One farmer who used erythromycin in the footbath was unaware it was an antibiotic. Of farms that used bandages as part of lameness treatment (91% of farms), only 21% removed the bandage within three days. Cows were always re-examined after treatment on 11% of farms, were re-examined only if still lame on 71% of farms, and never re-examined on 18% of farms.

### Milking practices

The median distance cows walked on average from the paddocks to the collecting yard across all farms was 483 m (IQR = 300—600). The median distance to the furthest paddock from the collecting yard was 1000 m (IQR = 713—1200). Forty-four percent of farmers used a vehicle and 35% had a dog present when bringing cows in from the paddocks. Five percent used a backing gate to encourage cows into the parlour. The median holding time in the collecting yard for the last cow into milking was 80 min (IQR = 60—90). A quarter of farmers always held their cows after milking prior to returning to the paddock, 29% sometimes held their cows back, and on 46% of farms the cows always returned straight to their paddock.

The median space per cow in the collecting yard was 1.44 m^2^ (IQR = 1.14 – 1.88). Twenty-nine percent of farms had less than 1.20 m^2^ per cow (minimum recommended space per small cow; [37]) and 53% of farms had less than 1.5 m^2^ per cow (minimum recommended space per large cow; [37]). Twenty-four percent of collecting yards were predominantly smooth concrete, 30% predominantly grooved concrete and 30% predominantly slats. At the parlour entrance, 36% of farms had a step, 30% a slope, 31% a 90-degree turn and 8% a 180-degree turn. At the parlour exit, 26% of farms had a step, 23% a slope, 89% a 90-degree turn and 30% a 180-degree turn. The median distance cows had to turn after milking (first milking unit to the back wall) was 2.49 m (IQR = 1.89 – 3.16). No farms used rubber matting at the milking parlour exit.

### Cow tracks

Thirty-eight percent of farmers had added new cow tracks and 34% had renovated parts of their cow tracks in the last five years. Twenty-one percent of farmers aimed to repair their cow tracks at least once per year. Cow track widths and gradients are shown in Table 2 and track surface types in Table 3. Fifty-two percent of farms had at least one rough cow track and 9% had at least one very rough cow track in the first fifty metres following the collecting yard. Seventy-nine percent of farms had at least one cow track with a sharp turn, and 79% with an inconsistent width in the first fifty metres. Fifty-four percent of farms also had at least one cow track measurement recorded as rough and 5% very rough on the track in use on the day of the grazing visit.

**Table 2:** The median cow track and verge widths across 99 spring-calving, pasture-based farms. Measurements were taken fifty metres from the collecting yard on all cow tracks, and at the estimated half-way point between the collecting yard and pasture and the end-point of the cow track that was in use on the day of the grazing visit. The average gradient for the cow track in use and the gradient of the steepest slope within the first fifty metres are also reported

Cow track characteristic	Median (IQR)	
	First 50 m	Cow track in use
Average width (m)	4.31 (3.67 – 4.98)	3.68 (3.05 – 4.42)
Average verge width (m)	0.45 (0.26 – 0.61)	0.53 (0.40 – 0.67)
Average gradient (%)	n/a	4 (2 – 6)
Steepest gradient (%)	12 (7 – 17)	n/a

*n/a* not measured on-farm

**Table 3:** Percentage of farms with each surface material present within the first fifty metres of cow track following the collecting yard and on the cow track in use on the day of the grazing visit, from 99 spring-calving, pasture-based farms. For the cow track in use, surface material was recorded at the estimated half-way point between the collecting yard and the paddock and the end-point of this cow track

Cow track surface material	Farms (%)	
	First 50 m	Cow track in use
Subsoil	83	91
Concrete (smooth, grooved)	70	38
Concrete slats	26	1
Stones/gravel	19	18
Earthen (grass/soil)	7	42
Tarmac	5	1
Astro-turf	1	0

### Paddock gateways

The median gateway width across farms, for the gateway in use on the day of the grazing visit, was 6.27 m (IQR = 5.06 – 7.96). Only nine percent of gateways were narrower than the cow track. Seventy-six percent of gateways had earth (grass/soil) as part of the gateway surface, 38% subsoil and 19% stones. Across farms, 46% of gateways measured were rough, and 8% very rough.

### Winter housing

All farms used cubicle housing and 10% had additional loose housing (straw yards and slatted pens). Considering all housing types, 6% of farms had at least 0.6 m (recommended feeding space; [38]) available per cow at the feed barrier in all pens; in contrast, 58% of farms had less than 0.6 m available in all pens. Thirty-six percent had a combination of pens with and without 0.6 m per cow available at the feed barrier. Across farms, the median of the average feed space per cow across pens was 0.49 m (IQR = 0.40 – 0.60). Fifty-six percent of farms had dead-ends present in all pens, 5% had no dead-ends present in all pens and 39% had a combination of pens with and without dead-ends. Seventy-one percent of farms had grooved concrete present within the housing environment, 65% smooth concrete and 1% concrete flooring with rubber mats. In addition, 86% of farms had smooth concrete slats within the housing environment, 14% grooved concrete slats, and 5% slats with rubber matting.

For cubicle housing, 15% of farms had at least 1.1 cubicles per cow (recommended best practice; [39, 40]) in all pens, 56% of farms had less than 1.1 cubicles per cow in all pens and 28% had a combination of pens with and without 1.1 cubicles per cow. Across farms, the median of the average number of cubicles per cow across pens was one (IQR = 0.92 – 1.07). On 6% of farms cubicles had no mats or bedding present and cows were lying on concrete bases only; a further 6% of farms had a combination of cubicles with and without mats or bedding. The remaining 88% of farms had mats or bedding present on all cubicles. On 69% of farms, cubicles were in very good (< 5% in disrepair) or good (5–24% in disrepair) condition in all pens. On 5% of farms, cubicles were in poor (25–50% in disrepair) or bad (> 50% in disrepair) condition in all pens. On 14% of farms, there were a combination of pens with very good/good cubicle condition and poor/bad cubicle condition. Eight percent of farms had a brisket board present on all cubicles measured, 64% had no brisket board present on all cubicles measured, and 15% had a combination of cubicles with and without a brisket board. Fifty-nine percent of farms had a neckrail present on all cubicles measured, 3% had no neckrail present on all cubicles measured, and 23% had a combination of cubicles with and without a neckrail. Details on cubicle dimensions can be viewed in Table 4.

**Table 4:** Median cubicle dimensions across 85 spring-calving, pasture-based farms

Average cubicle dimensions (m)^a^	Median (IQR)
Curb height^b^	0.24 (0.22 – 0.25)
Width^c^	1.10 (1.07 – 1.12)
Neckrail height^d^	1.10 (1.06 – 1.12)
Diagonal length^e^	2.00 (1.96 – 2.05)
Bed length^f^	1.72 (1.68 – 1.87)
Lunge space^g^ (wall facing cubicles)	0.59 (0.51 – 0.67)
Lunge space^g^ (head to head cubicles)	0.54 (0.47 – 0.62)
Total length^h^ (wall facing cubicles)	2.18 (2.12 – 2.26)
Total length^h^ (head to head cubicles)	2.14 (2.09 – 2.25)

^a^A proportion of cubicles in each pen were measured (5% of the two most common cubicle types, with a minimum of two cubicles per type)

^b^From pen floor to upper surface of cubicle

^c^Between inner edges of cubicle partition at cubicle entrance

^d^Bottom of neckrail to surface of cubicle (only recorded if neckrail present)

^e^Back edge of cubicle to near-side of neckrail (only recorded if neckrail present)

^f^Back edge of cubicle to base of brisket board (only recorded if brisket board present)

^g^Front of neckrail to wall or mid-way between cubicles (only recorded if neckrail present)

^h^Back edge of cubicle to wall, or to midpoint between head-head cubicles

## Discussion

The median herd-level lameness prevalence was 7.9% during the grazing period and 9.1% during the housing period; which was comparatively lower than that commonly reported in cattle in fully housed systems [19, 41, 42]. Average herd-level lameness prevalence in fully housed systems has previously been reported at 55% in the North-Eastern U.S. [19], 39% in the UK [41], 36% in Austria [42], 31% in California [19], 28% in British Columbia [19], 25% in Minnesota [43] and 21% in Québec, Ontario, and Alberta [44]. It is possible that the long grazing periods contributed to reduced lameness during the housed period. Access to pasture has been shown to reduce lameness prevalence [41] and risk of hoof disorders [45]. Lameness prevalence in the current study was lower than Somers et al. [26] who reported prevalence in Irish pasture-based systems to be 11.6% before and after breeding, with an escalation to 14.6% during breeding. The higher prevalence reported by Somers et al. [26] may be due to differences in farm location, management practices and lameness scoring time frame (February to August only). Lameness data was also only recorded on ten farms. O’Connor et al. [25] reported herd-level lameness prevalence in Ireland to be 11% early in the grazing season and 5.9% later in the grazing season. Ireland’s pasture-based dairy system is considered to be beneficial for dairy cow welfare; maintaining this positive reputation provides a marketing advantage for Irish dairy produce. The lameness prevalence reported in this study compares well with other nations and could, therefore, strengthen the competitive and sustainable nature of Irish agriculture.

Although lameness prevalence in Irish pasture-based systems was shown to be comparatively low compared to fully housed systems, approximately forty percent of cows that were lame at grazing were also lame when scored at housing, which is clearly a welfare concern. However, as lameness scoring in this study occurred at two time points only, this may be due to reoccurring lameness as opposed to a single continuous lameness event. Scoring twice per year only may also miss the impact of seasonality on lameness. For example, it may be expected that lameness could peak towards the end of the housing period and into the start of the grazing period. A follow-up study monitoring the changes in lameness over a full lactation, through regular and frequent lameness scoring, would further help with understanding the dynamics of lameness in a pasture-based system.

It has been previously demonstrated in a longitudinal study that a history of lameness is a risk factor for a future case of lameness [46]. To prevent cows becoming chronically lame, early detection and treatment is vital [29, 30]. Only a single farm in this study performed lameness scoring to detect lame cows, and even more surprisingly, over half of farmers were not familiar with the concept of lameness scoring. In the UK, it is recommended that lameness scoring is carried out at least once per month, to enable early detection and allow producers to benchmark against other herds and within their own herd [34]. Good lameness detection on a daily basis by trained staff is also critical for detecting and treating lame cows promptly. Approximately a quarter of Irish dairy farmers waited over two days before treating a cow that was identified as lame. Twenty-one percent of farmers also waited for more than one cow to be identified as lame before treating. Given the relatively low lameness prevalence, this could lead to a very long period of time between detection and treatment, which could possibly explain the high number of reoccurring cases found in this study. These results suggest there is huge scope for improving lameness management on Irish dairy farms, through providing information and guidance on detection and early treatment of lameness.

Although early detection and treatment is vital for ensuring recovery of lame cows, lameness prevention strategies are critical to reduce lameness in the first instance. Routine trimming of the entire herd, as a method to prevent lameness, was uncommon on Irish pasture-based herds; six percent of farmers carried out this practice, which was lower than the fourteen percent of farmers that reported routine trimming in 2015 [47]. However, routine trimming may not be as important for cows in grazing herds due to wear on the hoof from walking long distances between the milking parlour and the paddocks; cows in this study were on average walking between 1200 and 2400 m per day. Routine trimming can also be a useful method for early detection of mild lesions and correcting overgrown claws, thereby preventing future lameness cases [48, 49]. Further research is required to determine if routine trimming in a pasture-based system is beneficial and economically viable.

Footbathing is another approach to help reduce lameness at herd-level, by treating and preventing the infectious disease digital dermatitis [50]. The presence of digital dermatitis in a herd (according to the farmer), has been found to be predictive of lameness [33]. Forty-four percent of farmers reported having digital dermatitis in their herd; however, only 31% of farmers footbath more than twelve times per year. Based on a meta-analysis, Jacobs et al. [51] reported that footbathing at least four times a week with 5% copper sulphate was the only protocol that showed a reduction in digital dermatitis compared to control groups (no footbath or water footbath). There are, however, limited guidelines on the optimum footbathing frequency and product for pasture-based herds; further research is required in this area. It must also be noted that the use of copper sulphate for footbathing is currently illegal under the EU biocide regulations [52]. O’Connor et al. [25] reported an association between footbathing and lameness in Irish pasture-based dairy herds; however, this is likely due to farmers deciding to footbath if they have a lameness problem in their herd. It is also recommended that the footbathing solution is changed after 100 to 300 cows [53]. This protocol was followed by the majority of farmers in this study; however, only twenty percent of footbaths were at least three metres long, which is the recommended length to allow for two immersions of each hind hoof [53].

A herd health plan should outline farm-specific management practices to help improve dairy cow health, whilst maintaining a productive herd. A herd-health plan should be continuously updated as management practices are implemented and the health of the herd reviewed [54]. A herd heath plan requires a team approach with the farmer and the farm's veterinarian. Only fifteen percent of farmers in this study had a herd-health plan which incorporated lameness protocols. As part of the Sustainable Dairy Assurance Scheme [55] in Ireland, farmers are only required to report in brief the months of the year they plan to check and treat lameness. In contrast, UK dairy farmers are required to have a detailed lameness herd health plan, reviewed by a veterinary professional, as part of the Red Tractor farm assurance scheme [56]. Keeping accurate records of detected lame cows is also an essential tool for monitoring individual cows and providing herd-level information [57]. Keeping records will help establish if a cow has a recurring or first-time lameness case, enable farmers to monitor problem cows and establish the main causes of disease. In this study, only one-fifth of farmers kept records of lame cows detected in their herd, which demonstrates that there is an urgent need for improved communication to farmers regarding the benefits of record keeping.

The use of antibiotics as a footbathing solution is not currently licensed in Ireland [58]; however, nine percent of farmers still reported using antibiotic footbaths as a treatment for digital dermatitis. Even more worryingly, one producer did not know that the product they were using was an antibiotic. Continued use of antibiotic footbaths presents a global health risk due to antimicrobial resistance [59]. Bell et al. [60] also reported that antibiotic footbaths only relieved digital dermatitis symptoms for a short duration. In the current study, farmers favoured injectable antibiotics over pain-relief to treat lameness; a very low proportion of dairy farmers in Ireland provided pain relief to severely lame cows. Implementing pain management will dramatically improve cow welfare and improve recovery rates; Thomas et al. [29] reported that a therapeutic trim followed by a block placed on the sound claw, in conjunction with non-steroidal anti-inflammatory drugs (NSAIDs), improved the cure rate of lameness by 16% compared to cows that only received a therapeutic trim. Kasiora et al. [61] also showed that freshly calved lame cows that were given a singular dose of ketoprofen produced 10.49 kg more milk per day than the control group. Lame cows also benefit from being in close proximity to the milking parlour to reduce the distance they have to walk; Thomsen et al. [62] reported that housing lame cows in a hospital pen improved recovery compared to lame cows housed with the entire herd. However, only twelve percent of farmers in this study put severely lame cows in a closer paddock. There is an immediate need to provide information to farmers regarding the appropriate treatments for lame cows, and especially the importance of pain-relief.

There are various views on the use of bandages for the treatment of hoof lesions. Klawitter et al. [63] reported that the use of topical treatment and applying a bandage to M2 digital dermatitis lesions for four weeks, changing the bandage on a weekly basis, increased the cure rate compared to lesions that only received the topical treatment. In contrast, a recent study reported that sole ulcers were less likely to heal following treatment when a bandage was applied [64]. However, a bandage may be beneficial for severe cases when the corium is considerably exposed or when the lesion is excessively bleeding [65]. A bandage can improve cleanliness and prolong contact with the topical treatment; however, leaving a bandage on for a significant length of time can lead to contamination from manure [63], preventing lesions from healing. In the current study, only twenty-one percent of farmers who used bandages removed the bandage within three days following application. Farmers who do not actively take responsibility to ensure bandages are removed promptly, should avoid having bandages applied to lame cow by either themselves or the hoof trimmer [65].

The milking routine can impact the risk of lameness in dairy cows; prolonged standing at milking can compromise the time budget by reducing lying times and feeding times [66], increase the risk of lameness, and negatively impact animal welfare [67, 68]. In this study, the median holding time for the last cow into milking was 80 min, which is comparable to a milking time of 83 min in Australian pasture-based systems for herd sizes of less than 150 cows. However, Beggs et al. [69] also reported that milking time increased to over 2.5 h in larger herds. If herd expansion continues, farmers must improve milking efficiency or consider having separate milking groups to prevent an increase in standing time on concrete collecting yards, which increases the risk of lameness [68]. A quarter of farmers in this study also held back their cows following every milking without access to cubicles or a lying area, instead of allowing them to return straight back to the paddock. This results in cows spending more time away from the paddock and standing on hard concrete surfaces for longer. An increase in the time cows spent away from their pen due to milking was previously associated with increased lameness prevalence [67]. It was speculated that this was due to the negative influence on lying time.

On the majority of farms in this study, cows were required to make a sharp turn at the parlour exit. The median distance available for cows to make a turn (first milking unit to back wall) was 2.49 m; which is only the approximate body length of a dairy cow. Previous risk factor analysis (as part of this same project) found that a shorter distance to turn at the parlour exit imposed a risk of lameness [33]. Sharp turns may reduce cow-flow and increase shearing forces on the hooves [70]. No farms in this study used rubber matting at the parlour exit, despite the high number of parlours with sharp turns. Rubber matting has been shown to reduce slipperiness and improve mobility [71], and may therefore be beneficial at the parlour exit, particularly if the distance available for cows to make a turn is short.

Well-designed and maintained cow tracks can be very beneficial in reducing the risk of lameness for dairy cows in a pasture-based system [72]. According to Irish government guidelines [73], the median cow track width recorded in this study (3.68 m; cow track in use on the day of the grazing visit) is suitable for a maximum herd size of 68 cows. However, the median herd size in this study was 116 cows. This provides evidence that on a large number of farms, cow tracks were too narrow and would benefit from widening to prevent pushing and overcrowding of cows. It is theorised that this pushing results in shearing forces on the hooves and prevents cows choosing their preferred hoof placement to avoid stones. The majority of farms also had at least one cow track of inconsistent width in close proximity to the collecting yard; this may lead to a bottleneck, reducing cow flow and posing a risk of lameness [74]. In contrast, on most farms the paddock gateway measured was at least the width of the track, which enhances cow-flow as cows enter the paddock.

Rough cow tracks are a major contributing factor to lameness. It is speculated that rough surfaces can cause shearing forces on the hooves and may lead to separation of the white line due to loose stones penetrating the sole of the hoof. Over half of farms in this study had at least one cow track measurement that was classified as rough or very rough. Harris et al. [75] stated that a fine track surface material with no broken sections would help minimise lameness incidence. On over half of farms in this study, the gateway measured was also rough or very rough. Recent findings have shown that a ten percent increase in the proportion of stones as the gateway surface material, increased the risk of lameness by seven percent [33]. This study demonstrated that improving cow track conditions on farms is likely very important to reduce lameness prevalence.

In a part-housed, part-grazed system, farmers may not prioritise investment in housing facilities because cows are only housed for a short period of time compared to a fully housed system. It was previously reported that there was no difference in investment in housing infrastructure between Irish dairy farmers who expanded and those that did not. Investment was primarily focused on milking facilities in expanding herds [31]. Although the majority of farmers in this study used bedding or matting on all cubicles, on 12% of farms, all or a proportion of cubicle beds were bare concrete. Also, only 15% of farms had at least ten percent more cubicles than cows in all pens; which is the recommended best practice for dairy herds [39]. Poor cow comfort and overstocking of cubicles can discourage lying behaviour [76, 77], which is a predisposing risk for lameness [78]. Farmers must be cautious of expanding their herd without increasing the space available in the housing environment.

## Conclusion

This study found that the majority of farmers were not familiar with lameness scoring and did not lameness score their herd. Routine trimming and footbathing was also not regularly undertaken and cows were not treated promptly enough. The use of NSAIDs to treat lame cows and putting lame cows in a paddock close to the parlour were not common. Most farmers did not keep records of lame cows or have a lameness herd health plan. The majority of farms had rough and narrow cow tracks, a proportion of farms had bare concrete cubicles (no matting or bedding) and the majority of famers had less than 1.1 cubicles per cow. Irish dairy farmers appear to lack knowledge of the key practices and environment necessary to ensure low levels of lameness. There is an urgent need to provide farmers with more information and guidance on how to improve management and infrastructure to reduce lameness risk and improve dairy cow welfare.

## Data Availability

The datasets used and analysed during the current study are available from the corresponding author on reasonable request.

## Abbreviations

IQR: Interquartile range
LS: Lameness score
NSAIDs: Non-steroidal anti-inflammatory drugs
